# Relationships of Maternal Employment and Work Impact with Weight-Related Behaviors and Home Environments of Mothers and Their School-Age Children

**DOI:** 10.3390/ijerph20146390

**Published:** 2023-07-18

**Authors:** Elena Santiago, Virginia Quick, Melissa Olfert, Carol Byrd-Bredbenner

**Affiliations:** 1 Maryland SNAP-Ed Department, Family and Consumer Sciences, University of Maryland, Columbia, MD 21044, USA; elena1@umd.edu; 2 Department of Nutritional Sciences, Rutgers University, New Brunswick, NJ 08901-8520, USA; vquick@njaes.rutgers.edu; 3 Department of Animal and Nutritional Sciences, University of West Virginia, Morgantown, WV 26506-3740, USA; melissa.olfert@mail.wvu.edu

**Keywords:** working mothers, maternal employment, work gains, work strains, work impact, weight-related behaviors, home environment, weight status

## Abstract

The prevalence of obesity continues to rise. Preventing obesity, especially childhood obesity, is critically important. Parents, especially mothers, play a vital role in preventing childhood obesity. Numerous factors, such as maternal employment, may influence maternal weight-related practices and home environment characteristics that affect the risk of childhood obesity. Given the prevalence of both childhood obesity and maternal employment, this study was conducted to examine how weight-related maternal, child, and household behaviors as well as home environment characteristics differ by maternal employment hours and extends existing research by examining work impact on behaviors and home characteristics. U.S. mothers (n = 527) with at least one school-age child (6 to 11 years), who were between the ages of 25 and 54 years and the main food gatekeeper in the household completed an online survey. ANOVA comparisons of non-working, part-time employed, and full-time employed mothers revealed few differences in any of the variables studied. Cluster analysis of the 336 employed mothers based on six work impact scale scores found three unique clusters characterized as Enthusiastic Earners, Indifferent Earners, and Strained Earners. Few differences in sociodemographic and job characteristics occurred among clusters and the differences noted had small effect sizes. Clusters did not differ by maternal BMI or perceived child weight status. However, the clusters differed in numerous weight-related behaviors and home environment characteristics. Future research should aim to determine the direction of the associations of work impact with weight-related behaviors and home environments as well as identify potential strategies for overcoming the negative effects of employment on weight-related behaviors and environments and weight status as well as clarify other factors that may affect maternal work impact, such as time management, reasons for employment, and stress.

## 1. Introduction

The obesity epidemic is an ongoing global concern [1]. Approximately 42% of adults and nearly 20% of children and adolescents between the ages of 2 and 19 years in the United States have body mass indexes (BMI) categorized as obese [2]. Those with obesity in childhood bear the future burden of having an increased risk of remaining obese into adulthood, which can lead to long-term health consequences [3,4]. Along with physical health risks, children and adults with obesity are more likely to experience higher rates of social stigmatization and mental health conditions such as depression [5,6,7].

Prevention of obesity, especially childhood obesity, is critically important [8]. Parents play a vital role in reducing the risk of childhood obesity, in that they serve as role models for their children, exerting a major influence on shaping children’s weight-related behaviors, such as eating, exercising, and sleeping [9]. For example, parents make daily decisions about foods that will be eaten in the home, facilitate or deter opportunities for sedentary and active play, and set and enforce bedtimes or not—all of which can influence their children’s dietary intake, physical activity level, sleep duration and, ultimately, their body weights. Parents also are largely responsible for creating the home environment where weight-related behaviors are performed, such as providing space and support for physical activity and supplying food for the home. Thus, parental influences range from the individual level (e.g., their own behaviors and cognitions) to the home environment (e.g., active play toys and games, settings for good quality sleep).

Despite the move toward greater involvement of fathers in parenting responsibilities, mothers remain the primary child caregiver and food gatekeeper in most households [10,11,12,13] and are, therefore, an important audience to study. Factors that may influence maternal weight-related lifestyle practices and home environments include personal preferences, experiences, and values, as well as education, income, management skills, and health [14]. Research suggests that maternal employment is another factor that may influence household weight-related lifestyle practices, home environment characteristics, and childhood obesity risk [15,16,17]. For instance, maternal employment may affect lifestyle patterns and responsibilities (i.e., less preparation time for family meals at home, more carry-out meals), and time and energy available to spend on parenting and household tasks [16,18,19,20]. Some studies indicate that children with working mothers are less physically active, spend more time in sedentary behaviors (e.g., watching television), and eat less healthy diets [14,15,17,21]. Employment-related stressors may have negative impacts on both parent and child weight-related behaviors [22,23,24,25]. Examples of work stressors include performance demands, job security, work time spillovers into home life to complete work-related tasks, motivation and commitment to be more productive in career goals, and pressures caused by workload and/or other employees [22,23,24,26]. Although limited attention is given to the positive impact of employment on family life and work, several benefits of maternal work have been recognized (e.g., increased household income, prestige of work promotions, possible personal growth and development within the work organization) [25]. Similarly, when mothers are employed, home quality improves and children learn good work ethic values [25]. Working also may improve maternal perceptions of parenting abilities and affords mothers the ability to be involved in other activities or interests outside of the home environment [25].

In 2020, 71% of women in the United States with a child under the age of 18 years participated in the workforce [27,28]. Although the maternal employment rate has increased steeply over the past 5 decades, there continues to be limited research examining the relationships between maternal employment and weight-related behaviors and home environments [28,29]. The studies that do exist have shown that job characteristics, such as maternal hours worked and non-standard shifts, are linked with higher child weight status [16,17,30,31,32]. Studies tend to focus on total hours worked, with little attention to the links between the impact of work (positive and negative effects) on mothers and family weight-related behaviors and home environment characteristics [16,17,20,32]. Given the prevalence of both childhood obesity and maternal employment, especially the employment of mothers with school-age children [33], this study was conducted to examine how weight-related maternal, child, and household behaviors, as well as home environment characteristics, differ by maternal employment hours, and it extends existing research by examining work impact on behaviors and home characteristics.

## 2. Materials and Methods

This study was approved by the Institutional Review Board at Rutgers University (Protocol #2020001192). All participants gave their informed consent online. Data were collected via self-report survey conducted online between March 20 and 28 2020.

### 2.1. Sample

Dynata, an online market research firm, electronically recruited participants for the survey. Participants were initially screened for eligibility criteria by Dynata using their database of participant characteristics, and then eligibility was confirmed at the start of the survey administration [34]. Survey participants received a modest stipend (approximately USD 10) from Dynata in the form of cash, points, prizes, or charitable donations.

The eligibility criteria for this part of a multicomponent study included being a mother who had at least one school-age child (6 to 11 years), was between the ages of 25 and 54 years, was the main food gatekeeper in the household (i.e., made most or all food purchasing and preparation decisions), was able to read English, and resided in the United States. Using the 2020 U.S. Census data, a total sample size of at least 385 was needed, based on the total population of women in the U.S. between the ages of 25 and 54 years old, with a 95% confidence interval to ensure that the characteristics of the population were accurately estimated by the sample survey with a 5% margin of error [28,35].

### 2.2. Survey Instrument

The “Home Obesogenicity Measure of EnvironmentS-2” (HOMES-2) survey was used to collect data online [36]. This survey instrument assessed maternal sociodemographic characteristics, maternal and child weight status, weight-related maternal, child, and household behaviors, and home environment characteristics. This survey also gathered data on maternal employment characteristics and work impact. This survey is described in great detail elsewhere (see reference [36], Supplementary table 2); a summary of the survey scales follows.

Sociodemographic characteristics. The sociodemographic characteristic data collected included the mother’s age, race/ethnicity, highest level of education, and the number of parents and children in the household. Mothers also reported hours/week spent on household, parenting, and community activities. If a spouse or partner was present in the household, spouse employment status and education level were collected.

Weight status. Self-reported heights and weights were used to calculate the BMI of the mothers. The mothers indicated the body shape of their school-age child using the Collins scale, which consists of a series of 7 age-appropriate, sex-specific silhouettes ranging from very underweight to very obese [37]. For households with more than one child in this age range, mothers were instructed to report on the child born closest to a randomly selected and specified time and date.

Weight-related behaviors. This section of the survey measured physical activity levels, nightly sleep duration, and dietary intake of mothers and children. Physical activity was assessed using the Streamlined, Enhanced Self-Report Physical Activity Measure [38]. This measure determines days/week of participation in vigorous physical activities, moderate physical activities, walking, and resistance training, with days weighted based on the intensity of activity (i.e., 3, 2, 1, and 1, respectively) and then summed [38]. Thus, total scores could range from 0 to 49, with higher scores indicating greater levels of physical activity. The sleep duration component of the Pittsburgh Sleep Quality Index determined hours of sleep nightly [39,40]. The 7-item Block Fruit-Vegetable Screener determined daily servings of fruits and vegetables consumed [41,42]. The 6-item HOMES-2 Sugar-Sweetened Beverages item estimated servings of these drinks consumed daily (e.g., soft drinks, fruit drinks, sports drinks) [36].

Household behaviors focused on family meal practices. Mothers reported the total number of family meals eaten weekly, the days/week these meals were eaten while simultaneously using electronic media devices, and the days/week family meals were eaten in unhealthy locations (i.e., in the car, at convenience stores, or at fast food restaurants) [36].

Home environment. This section of the survey gathered data on physical activity space and supports, sleep supports, and household food availability. The HOP-Up questionnaire for households with school-age children assessed the physical activity space and supports inside the home (3 items) and in the area outside the home/yard (2-items) [36,43]. An indicator item assessed sleeps supports (i.e., comfortable, dark, quiet place to sleep) [36]. Household availability of fruits and vegetables was assessed with the Block Fruit-Vegetable Home Availability Screener, which estimates the servings of fruits and vegetables available per person in the household per day [41,42]. The HOMES Sugar-Sweetened Beverage Home Availability item measured the servings of sugar-sweetened beverages available per person per day [36].

Maternal employment. This section of the survey gathered descriptive information on the total number of paid employment jobs worked per week, total days per week usually worked, days/week worked outside the home, total hours of paid employment per week, extra hours worked to complete job-related duties, weekend days worked, work schedule regularity (i.e., same days and hours worked each week), typical work start and finish times each day, daily commute time, and determination of pay (i.e., set salary or per time worked).

This section of the survey also included 6 scales assessing work impact. Response choices were scored on a 5-point Likert type scale (strongly disagree, disagree, neutral, agree, strongly agree) from 1 to 5, respectively. The scale items were averaged with higher mean scores indicating greater expression of the characteristic evaluated. The 3-item Work Motivation scale assessed the degree to which the mother felt that the work she did was appreciated, interesting, and had a secure future [24]. Work Involvement was assessed with 1 item focused on the extent to which mothers were engaged with their work for pay, with high scores indicating they “ate, drank, and breathed their work” [44]. Scales from the Work-Family Balance and Family Relationships Scale [45] assessed mothers’ perceptions of positive and negative effects on family responsibilities and parenting when combined with their work for pay [45]. The 7-item Work-Family Strains Scale assessed mothers’ perceptions of negative effects, or “strains”, on family responsibilities as a result of their work for pay whereas the 7-item Work-Family Gains Scale assessed mothers’ perceptions of positive effects, or “gains”, on family responsibilities when combined with their work for pay. The 6-item Work-Parenting Strains Scale assessed mothers’ perceptions of strains on parenting caused by their work for pay and the 4-item Work-Parenting Gains Scale assessed mothers’ perceptions of gains in parenting when combined with their work for pay. 

### 2.3. Data Analysis

All analyses were conducted using JMP^®^ Pro 16.1.0 (SAS Institute; Cary, NC, USA). Mothers were assigned to employment categories based on paid employment hours/week (i.e., no hours of work for pay, part-time (<30 h/week), or full-time (≥30 h/week)) [46]. ANOVA and Tukey post-hoc tests were conducted to determine differences in behavior and home environment variables among and between maternal employment categories.

Next, mothers who worked for pay were partitioned into work impact groups using a multistep cluster analysis process of the 6 work impact scales (i.e., Work Motivation, Work Involvement, Work–Family Strains, Work–Parenting Strains, Work–Family Gains, Work–Parenting Gains [24,44,45]). Cluster analysis merges individuals into groups that maximize between-group heterogeneity and within-group homogeneity. The first step of this analysis involved conducting Ward’s hierarchical cluster analysis procedures using the work impact scales to identify the ideal number of clusters by examining the scree plot and agglomeration schedule [47,48]. Ward’s hierarchal cluster analysis was the preferred method for the first step in the cluster analysis, because the ideal number of clusters was not known a priori, and this analytic process is well suited to moderate sample sizes (i.e., less than 1000) [47,48]. The second step was to conduct k-means cluster analysis by specifying the ideal number of clusters identified by the Ward’s analysis, followed by analysis of variance (ANOVA) and Tukey post hoc procedures for all pairwise comparisons of the work impact scales. Step three established cluster stability by repeating step two 11 times (3% of the sample), each time using a unique, random half of the sample. Cohen’s kappa was conducted to establish cluster stability by comparing the agreement between the cluster assignment generated in step two with the assignment of each random half in step three. Differences in behavior and home environment variables among and between work impact clusters were investigated using ANOVA and Tukey post-hoc test.

The probability level for all ANOVA main effects was set at *p* ≤ 0.01 to reduce the risk of type I errors due to the multiple comparisons made. Post hoc probability for pairwise comparisons was set at *p* ≤ 0.05. Partial eta-squared values were calculated to determine the effect size of all ANOVA main effects meeting the threshold for significance [49]. Values of 0.01, 0.06, and 0.14 indicate small, medium, and large effect sizes, respectively [49].

## 3. Results

Of the 1342 mothers recruited to take the online survey, 815 were not included because they did not meet the eligibility criteria, did not consent, did not complete the survey, and/or did not meet survey data quality checks. The final sample was 527 mothers.

### 3.1. Comparison of Mothers by Employment Status

As shown in Table 1, of the 527 mothers, 36% were not employed for pay, 20% were employed part-time and 44% were employed full-time. Mothers were about 37 years old with most being non-White, with neither of these characteristics varying by employment status. Mothers had an education level indicating at least some post-secondary education and averaged 2 to 3 children per household. ANOVA revealed that mothers who did not work for pay had a significantly lower education level and more children than those who worked for pay, with medium to large effect sizes. Most households had 2 parents, with an employed spouse or partner. Spouse/partner education level indicated most had at least some post-secondary education, with spouses/partners of mothers who did not work for pay having significantly less education than those of full-time working mothers; however, effect sizes were small. Mothers reported spending about 3 or 4h each day in household, parenting, and community activities, with time spent on these activities being significantly higher in non-employed mothers with a medium effect size.

Table 2 shows that behaviors, environmental characteristics, and weight status varied little by maternal employment status. Mothers in all employment categories had low physical activity levels, slept about 7 h per night, ate approximately 4 to 5 servings of fruits/vegetables daily, and drank about 1.5 servings of sugar-sweetened drinks per day. Their children also had low physical activity levels, slept for about 9 h nightly, ate 4 to 5 servings/day of fruits/vegetables, and had one sugar-sweetened beverage serving daily. Mothers reported having about two family meals daily, with mothers not working for pay reporting significantly more family meals weekly than those working full-time; however, the effect sizes were small. Each week, about two family meals were eaten in unhealthy locations, and electronic media were used during family meals about three times per week. Home environments were supportive of healthy physical activity, sleep, fruit/vegetable intake, and family meal behaviors. However, sugar-sweetened beverage availability was about three servings per household member per day. Maternal BMI was in the overweight range, and mothers perceived their children’s weight status to be slightly below the scale’s mid-point of healthy weight.

### 3.2. Comparison of Employed Mothers by Work Impact

The 336 study participants who were employed for pay were further analyzed to examine the effect of work impact on weight-related behaviors and home environments. Ward’s hierarchical cluster analysis procedures using the six work impact scales generated a scree plot and agglomeration schedule that identified stage 333 as the point at which the difference between the agglomerative coefficients increased sharply indicating a three-cluster solution (i.e., 336 mothers-333 mothers) as ideal. The three-cluster k-means analysis, followed by ANOVA, indicated that the mean scores of all six work impact scales differed significantly, with large effect sizes (Table 3). Tukey post-hoc tests revealed that all pairwise comparisons of the clusters and six work impact scales differed significantly which is indicative of unique cluster groupings. Cohen’s kappa coefficients comparing the original k-means clusters generated in step two of the cluster analytic procedure with the random halves in step three of this procedure averaged 0.84 ± 0.06SD (range=0.74 to 0.95)—a level of agreement considered “almost perfect” using the threshold criteria set by Landis and Koch [50] and indicative of cluster stability. 

The mean scores of the five-point clustering scales for Cluster 1 indicated high Work Motivation, Work–Family Gains, and Work–Parenting Gains, and low Work–Family and Work–Parenting Strains, along with moderate Work Involvement. Thus, this work-motivated, high-gain, low-strain, work–life balance cluster could be described as Enthusiastic Earners. Cluster 2 scored around the mid-point of the scale for all of the clustering variables, indicating that they were ambivalent about their Work Motivation, Work Involvement, and Work–Family and Work–Parenting Gains and Strains. Cluster 2 scored the lowest of all clusters on the Work Motivation, Work Involvement, and Work–Family and Work–Parenting Gain scales and, hence, could be described as Indifferent Earners. Cluster 3 had the highest Work Involvement and Work–Family and Work–Parenting Strains. They also had more Work Motivation and Work–Family and Work–Parent Gains than Indifferent Earners (Cluster 2) but less than Enthusiastic Earners (Cluster 1). Thus, Cluster 3 could be described as Strained Earners.

Table 4 compares the sociodemographic characteristics of each work impact cluster. ANOVA revealed that the mothers were typically in their late 30s, with Cluster 1 being significantly older than both other clusters; however, the effect size was small. Race/ethnicity data indicated that the clusters did not differ, and all clusters had a greater proportion of non-White participants than white participants. No differences were observed for education levels, with all clusters having at least some post-secondary education. Household composition was also similar across clusters, with households typically being composed of about two children and two parents. The time that mothers spent engaging in household, parenting, and community activities daily was similar across clusters. In households with a second parent (i.e., spouse/partner), nearly all of these family members were employed full-time and tended to have at least some post-secondary education.

Table 5 describes the characteristics of the mothers’ employment. Most mothers had one job, worked about 5 days per week, and worked most days at a location outside the home. The proportion whose pay was a set salary versus an hourly or daily rate was similar across clusters. The average hours per week was about 30, with Cluster 1 mothers working significantly more hours than other clusters, although the effect size was small. Those in Cluster 3 reported working significantly more extra hours outside the workplace catching up on work-related duties that could not be finished during the workday, with a small effect size. However, when total work hours and extra hours were combined, the clusters did not differ significantly. Commuting time, including time spent dropping off children along the way, was less than 2 h/week for Clusters 1 and 2, and about 2.5 h/week in Cluster 3, although the clusters did not differ significantly. Mothers in Cluster 2 were more likely to work on weekends than their counterparts, but the differences were not significant. The majority of the mothers had standard work schedules, working the same days each week, and starting and ending work at standard times.

Table 6 reports findings for the behavior, home environment, and weight status assessments. The maternal behavior findings indicate that, for all mothers, physical activity was low, nightly sleep averaged 7 h, the number of fruit/vegetable servings eaten daily was about four, and sugar-sweetened drinks averaged 1 to 2 servings per day. Cluster 2 mothers had significantly lower physical activity and fruit/vegetable intake than Cluster 3, with small effect sizes. Additionally, Cluster 3 drank more sugar-sweetened beverages than both other clusters, with small effect sizes.

In general, children’s behaviors tended to mirror maternal behaviors. Children of Cluster 3 mothers drank significantly more sugar-sweetened beverages than children of Cluster 1 and 2 mothers, with a moderate effect size. Sleep averaged 8 to 9 h/night, with children of Cluster 3 mothers sleeping significantly less, with a small effect size.

Regarding household behaviors, all clusters ate about two family meals daily. Cluster 3 ate significantly more family meals each week at unhealthy locations than both other clusters, with a medium effect size. Cluster 3 also used electronic media at mealtimes more frequently than Cluster 2, but the effect size was small.

Nearly all aspects of the home environment differed significantly between clusters. Cluster 1 had significantly more space and support for indoor and outdoor physical activity, as well as sleep supports, fruit/vegetable availability, and family meal space and supports, with small-to-medium effect sizes. Sugar-sweetened beverage availability did not differ across clusters.

Maternal BMI was slightly above the healthy weight range and did not differ by cluster. Mothers perceived that their children had healthy weights, with no differences between the clusters.

## 4. Discussion

The aim of this study was to examine the relationship of maternal employment status and work impact with weight-related behaviors and home environment characteristics. Comparisons of the sample of 527 mothers of children aged 6 to 11 years by employment status revealed few differences in any of the variables studied, other than education levels being lower, the number of children being higher, and the time spent engaged in household, parenting, and community activities being greater among mothers who did not work for pay than among those who worked. The subsample of the 336 employed mothers was separated into three unique clusters, whose work impact scores revealed that Clusters 1, 2, and 3 could be characterized as Enthusiastic Earners, Indifferent Earners, and Strained Earners, respectively. Few differences in sociodemographic and job characteristics were observed between clusters, and the differences that were noted had small effect sizes. The clusters did not differ by maternal BMI or perceived child weight status. However, the clusters differed in numerous weight-related behaviors and home environment characteristics.

The sociodemographic differences evident in the employment status comparisons are not surprising, given that education level and earnings tend to be positively correlated, and childcare tends to be costly [51,52]. Thus, it may make logical financial sense for less educated mothers (and, therefore, those with lower earning power) to stay home with their larger family than to seek outside employment and pay for childcare. Similarly, because they are not in the workplace, these non-employed mothers had more time for household, parenting, and community activities—surprisingly, this amounted to only about 70 to 90 extra minutes daily.

Employment status comparisons suggest that hours of paid employment play a minor role in weight-related behaviors, home environments, and weight status. These findings are contrary to reports that maternal employment can adversely affect children’s weight-related behaviors [16,18,20], such as physical activity and dietary intake—perhaps, as proposed by others, because children of working mothers have fewer opportunities for active play, spend more time watching television, have more unsupervised snacking, and eat fewer family meals [15,21,53]. This study’s findings are also incongruent with research reporting a greater risk of obesity in both working mothers and their children [16,17,20,30,31,32].

Mothers who work longer hours are more likely to face time constraints for completing weight-related self-care, parenting, and household responsibilities [15,21]. However, hours of employment had little relationship with the performance of these responsibilities, with the exception of family meals. Full-time working mothers in this and other studies [16,20,54] reported less frequent family meals than unemployed mothers, having about 1.5 fewer family meals each week. The fewer family meals and slightly more frequent consumption of meals in unhealthy locations (e.g., fast food restaurants) may account for some of the 1.25 to 1.5 h/day less time that employed mothers spent engaged in household, parenting, and community activities than their non-working counterparts. Employed mothers’ slightly higher physical activity contributed to some of this time difference. Maternal sleep also may account for some of this time difference, in that full-time mothers reported nightly sleep that was about 10 min longer than others. Another factor that may have offset the time differences between employed and unemployed mothers is their education level. Higher educational attainment is generally associated with healthier behaviors (e.g., physical activity, nutrient-dense foods) [18,53,55,56,57,58]. Greater educational attainment is also positively correlated with income, which confers greater access to housing with more availability of amenities (i.e., sidewalks, parks) that support physical activity and stores selling better-quality food [16,18]. The greater education levels of employed mothers may have helped them to develop better time-management skills, thereby enabling them to overcome work-related time constraints to ensure positive weight-related behaviors, home characteristics, and weight status comparable to their unemployed counterparts. Future study of how maternal education may protect health despite time constraints imposed by employment is warranted.

The total number of hours worked was generally unrelated to weight-related behaviors and home environment characteristics; however, this was not the case with work impact. Just as managing the demands of maternal employment can affect home life, home life can affect mothers’ professional work life and productivity [59,60]. For instance, mothers who have difficulty balancing work obligations and family life are more likely to be less satisfied and committed to their jobs and perform work duties more poorly [22,23,26,59]. The strains associated with work–family life balance often lead to burnout, with higher employee turnover rates, increased lateness, and greater employee conflict and/or irritation of other employees who have to pick up the workloads of stressed mothers [22,23,26,59]. These strains can also negatively affect weight-related behaviors, as demonstrated in this study and others [61,62,63]. For example, the most strained cluster (i.e., Strained Earners) had the least healthy household behaviors; these mothers and their children drank the most sugar-sweetened beverages and had food-related home environments that were less supportive of healthy weights. The source of the strains experienced by Strained Earners was not clear. Their sociodemographic characteristics paralleled those of other clusters. Certain job characteristics—such as working schedules (days and shifts) that vary often, nonstandard hours, weekends, and long commute times—are frequently cited as being negative impacts on work [18], yet these also did not differ significantly between clusters. It could be that the Strained Earners’ high levels of work involvement contributed to feelings of strain caused by the need to divert time from work to home-related responsibilities, such as family meals and stocking the home with healthy foods. Future research should aim to elucidate the factors beyond sociodemographic and job characteristics that contribute to the strains of Strained Earners and determine whether and how nutrition interventions might address them effectively (e.g., build skills to improve meal planning, grocery shopping, family meal preparation efficiency).

Time constraints (real or perceived) and/or time management skills may be an underlying reason for the differences observed between Enthusiastic Earners and both Strained Earners and Indifferent Earners. This supposition is supported by parallels between this study and previous research. Like the Strained Earners in this study, mothers with poor time management skills reported fewer family meals and more eating out [26,54,64,65,66,67]. Children of Strained Earners had the shortest sleep duration—a full 40 min short of the minimum recommendations for children in this age group (i.e., 9 to 12 h) [68]. Several studies indicate that poor time-management skills may be associated with maternal behaviors that shorten children’s sleep duration, such as permitting greater use of sedentary media by children or endorsing unstructured bedtimes [69,70]. Behaviors of Indifferent Earners are congruent with research reporting that mothers with poorer time-management skills tended to have lower supplies of nutrient-dense foods in the home due to not allocating time for planning meals and purchasing and preparing foods [70,71]. This inadequate allocation of time often contributes to reduced intakes of fruits and vegetables [70,71], as was observed in Indifferent Earner mothers in this study. Incorporating assessments of time management skills and priorities in future investigations would expand our understanding of how these skills are related to weight-protective behaviors and environments.

Although not explored in this study, the reason for paid employment may affect mothers’ feelings of work-induced strains or gains on family life. Meeting the financial needs of their family is a key reason that mothers seek paid employment. For some families, mothers must work to provide the essentials of food, clothing, and shelter [71,72,73]. In other families, maternal employment augments family income, helping to increase the family’s standard of living through greater purchasing power and/or benefits such as employer-provided health insurance [71,74]. Mothers whose families are dependent on their income for survival are more likely to experience greater emotional stress in adequately performing work and home duties [65,75], potentially leading to problems with balancing work and family [29] that manifest in less effort being given to household and parenting responsibilities. Evidence suggests that children of mothers who must work to support their family may experience negative impacts on weight-related behaviors, such as less physical activity, shorter sleep duration, more meal skipping, frequent eating on the go, and fewer meals at home [67,69,71,72,73,76]. Both Indifferent Earners and Strained Earners in this study shared many similarities with mothers who must work to support their family [16,63,64,65,67,69,71,72,73,74,77]. Additionally, the lower support for physical activity and sleep in the home environments of Indifferent Earners suggested that less income was available for health-supportive housing. The congruence of their behaviors and home environments with previous research tends to support the notion that one underlying cause for the differences between Enthusiastic Earners and both Indifferent Earners and Strained Earners may be financial concerns. An exploration of why mothers are employed and the relative burden of financially supporting their families may provide insights into the overall gain and strain felt by these earners.

In general, Enthusiastic Earners had healthier behaviors and home environments than the other clusters. Their high gain and motivation scores, coupled with low strain and moderate work involvement scores, suggest a positive outlook on life, along with less or perhaps better-managed stress. Having family meals more often, greater availability of fruits/vegetables, and more space and supports for physical activity and sleep align with previous studies of mothers with lower levels of stress [59,65,74,78,79]. Understanding why these mothers are able to achieve these healthy behaviors and environments could inform the development of interventions aiming to help working mothers manage stress and protect family health.

The similarity of the findings related to behaviors and home environments to previous research lends support to the idea that the underlying causes for the differences in employed mothers may have roots in financial, time management, and stress differences. Although this study was not designed to determine why the clusters of mothers differed, but rather how they differed, understanding “why” is an important goal for future research. This type of research could help nutrition and health intervention designers to determine how they might address factors that impair the ability of families to engage in healthy behaviors, such as by incorporating financial management, planned parenting responsibilities, time management, and/or stress management contents, or advocating for this type of training.

When examining the findings of this study, both strengths and limitations must be considered. As with any human research, this study was limited by participant self-selection. However, to promote a broader representation of mothers beyond those with an interest in nutrition, the recruitment materials for this study were written to use inclusive language designed to appeal to an array of mothers, such as “learn more about families”. The study participants also received compensation, which may have helped expand the likelihood of recruiting mothers with a range of interests.

Another strength was the sufficient power of the sample size, along with the sample’s general comparability to the overall population of women aged 25 to 54 years in the United States. The proportion of the sample that had a spouse or partner was comparable to that in the overall population of women in the sample’s age group (79% vs. 78%), but the sample had slightly more children than the U.S. average in 2020 (2.5 vs. 1.9) [80,81,82]. The mothers also had an average education level slightly higher than suggested by national data for women between the ages of 25 and 54 (mean education level of 2.3 vs. 2.1), with both attaining, on average, at least some post-secondary education [83]. The sampled mothers were demographically diverse and lived in regions spread across the United States, mirroring the United States population distribution [84]. The study participants were, however, more racially diverse than the U.S. population, in that 35% were white, compared to 55 to 60% nationally [85]. Although nearly two-thirds of the mothers in this study worked for pay, according to the United States Bureau of Labor, this rate is lower than the national average for mothers with a child under the age of 18 years (64% vs. 72%) [28,33,86]. The sample for this study was limited to only mothers and cannot be generalized to spouses/partners or other caregivers. The variations seen among the mothers (such as greater diversity, higher educational attainment, and lower employment rates than national statistics) may limit the generalizability of our findings to all mothers of children in the target age range. In addition, each mother reported on only one of their children in the school-age range, who may have differed from similar-aged siblings. For a more complete picture of family weight-related behaviors, future research should include spouses/partners and other children in the family. Additionally, collecting data from other child caregivers, such as babysitters, teachers, and coaches, could help to more fully characterize the weight-related behaviors of school-age children when they are away from home.

The cross-sectional nature of this study is another limitation, in that causality cannot be determined. However, the study’s findings can provide the basis for designing longitudinal research to explore causality and changes over time. Another limitation is that all data were self-reported. Thus, answers may have been biased due to social desirability and/or errors in memory [87]. However, online surveys can help reduce the risk of social desirability bias [88], because participants may be more likely to accurately answer questions that are sensitive in nature compared to participants in other survey data collection scenarios. Online data collection affords a sense of confidentiality and perceived anonymity that is not felt with data collection methods that are face-to-face [89]. In addition, a “preamble” statement was placed at the beginning of the survey to inform the participants that all responses were acceptable and that there were no right or wrong responses, so as to help reduce the likelihood of social desirability bias [89]. Online data collection offers many other advantages, such as ease of data collection and recording [90], along with increased ability to cost-effectively reach participants across the United States who would otherwise be difficult to access [88,89]. For participants, especially working mothers, online data collection is convenient.

Further strengths of the survey were that it consisted of valid and reliable scales and used strategies to reduce participant burden (e.g., grouping of related questions) and keep participants engaged (e.g., pictures, colorful fonts, virtual mental breaks). The survey data were thoroughly reviewed and cleaned to include participants who met all eligibility criteria and provided quality data.

An additional potential limitation is that data collection occurred in March 2020, soon after the World Health Organization declared COVID-19 a pandemic—a time when the earliest public health prevention measures were being implemented [90]. Furthermore, significant shifts in the labor market have occurred since our data collection, due to the COVID-19 pandemic. During the pandemic, more parents worked from home, and children attended school virtually; thus, parents were supervising and teaching their children while also working, which likely greatly influenced the number of hours worked and/or work impact [91]. This change in work schedules (including unexpected unemployment) and work location likely affected family health and weight-related behaviors. Furthermore, the COVID-19 pandemic affected some families more than others due to occupational status. For instance, parents with higher-paying jobs (e.g., doctors, lawyers) with more autonomy were affected less by the pandemic. However, occupational prestige among employed mothers was not considered in this study, which is an important topic for future research.

To the best of the authors’ knowledge, this is the first study to comprehensively examine the weight-related behaviors and home environments of mothers and their school-age children by maternal employment status and work impact. This study also moved beyond just examining total maternal hours of employment (an approach that has predominated maternal employment research [92]) to consider the impact of maternal work. The use of cluster analysis to group mothers by work impact and compare weight-related behaviors, as well as home environments, was novel, suggesting that factors beyond employment itself may affect maternal weight-related behaviors and home environments.

## 5. Conclusions

In conclusion, the findings of this study indicate that mothers of school-age children have diverse weight-related behaviors and home environments that seem to be associated not with hours of maternal employment but, rather, with the impact of work on mothers. Future research should aim to determine the direction of the associations of work impact with weight-related behaviors and home environments, as well as to identify potential strategies for overcoming the negative effects of employment on weight-related behaviors and environments and weight status that could be incorporated into obesity prevention interventions. In addition, future studies should also seek to clarify other factors that may affect maternal work impact, such as time management, reasons for employment, and stress.

## Figures and Tables

**Table 1:** Sociodemographic characteristics compared by maternal employment status.

Characteristic	Employment Status			ANOVA		Tukey’s Post Hoc Pairwise Comparisons for *p* ≤ 0.01 ^4^	Partial Eta-Squared
	**No Work for Pay**	**Part-Time** **(>0 to <30 h)**	**Full-Time** **(≥30 h)**	**F**	***p***		
	**n = 191**	**n = 106**	**n = 230**	**df = 2, 524**			
	**Mean ± SD**	**Mean ± SD**	**Mean ± SD**				
	**(95% CI)**	**(95% CI)**	**(95% CI)**				
Age (Years)	36.87 ± 5.61 (36.07–37.67)	36.93 ± 5.85 (35.81–38.06)	38.36 ± 5.92 (37.59–39.13)	4.16	0.0161		
Race/Ethnicity ^1^	0.68 ± 0.47 (0.61–0.75)	0.69 ± 0.47 (0.60–0.78)	0.61 ± 0.49 (0.55–0.68)	1.43	0.2405		
Education Level ^2^	1.98 ± 0.78 (1.87–2.10)	2.44 ± 0.65 (2.32–2.57)	2.53 ± 0.66 (2.45–2.62)	34.22	<0.0001	AB	0.116
# Children <18 Years in Household	3.37 ± 1.16 (3.21–3.54)	2.06 ± 1.07 (1.85–2.26)	2.06 ± 0.91 (1.94–2.17)	97.76	<0.0001	AB	0.271
# Parents in Household	1.80 ± 0.40 (1.74–1.86)	1.81 ± 0.39 (1.74–1.89)	1.76 ± 0.43 (1.71–1.82)	0.76	0.4687		
Maternal Household, Parenting, and Community Activities (Hours/Day)	4.39 ± 3.05 (3.96–4.83)	3.31 ± 1.82 (2.96–3.66)	3.13 ± 1.78 (2.89–3.36)	16.65	<0.0001	AB	0.060
Spouse/Partner	N = 153	N = 86	N = 175				
Spouse/Partner Employment ^3^	1.71 ± 0.65 (1.60–1.81)	1.63 ± 0.70 (1.48–1.78)	1.77 ± 0.60 (1.68–1.86)	1.35	0.2594		
Spouse/Partner Education Level ^2^	1.86 ± 0.86 (1.72–1.99)	2.03 ± 0.89 (1.84–2.23)	2.15 ± 0.87 (2.02–2.28)	4.79	0.0088	B	0.018

^1^ Coded as 0 = White; 1 = Non-White. ^2^ Coded as 1 = high school or less; 2 = some post-secondary education (e.g., college, technical school); 3 = baccalaureate degree or higher. ^3^ Coded as 0 = does not work for pay; 1 = works part-time for pay. 2 = works full-time for pay. ^4^ Significant pairwise comparisons: A = Clusters 1 and 2; B = Clusters 1 and 3; C = Clusters 2 and 3.

**Table 2:** Behavior, home environment, and weight status assessments compared by maternal employment status.

Assessment	Employment Status			ANOVA		Tukey Post-hoc Pairwise Comparisons for *p* ≤0.01 ^6^	Partial Eta Squared
	**No Work for Pay**	**Part-Time** **(>0 to <30 h)**	**Full-Time** **(≥30 h)**	**F**	***p***		
	**n = 191**	**n = 106**	**n = 230**	**df = 2, 524**			
	**Mean ± SD**	**Mean ± SD**	**Mean ± SD**				
	**(95% CI)**	**(95% CI)**	**(95% CI)**				
Maternal Behaviors							
Physical Activity Level ^1^	16.16 ± 12.44 (14.39–17.94)	19.16 ± 13.17 (16.62–21.70)	17.28 ± 12.88 (15.60–18.95)	1.8761	0.1542		
Sleep (Hours/Night)	7.10 ± 1.31 (6.91–7.28)	7.16 ± 1.17 (6.94–7.38)	7.22 ± 1.09 (7.08–7.36)	0.5773	0.5618		
Fruit/Vegetable Servings/Day	4.33 ± 2.02 (4.04–4.62)	4.52 ± 2.00 (4.14–4.91)	4.37 ± 2.16 (4.09–4.66)	0.2992	0.7415		
Sugar-Sweetened Drink Servings/Day	1.46 ± 1.97 (1.18–1.74)	1.50 ± 1.61 (1.19–1.81)	1.39 ± 2.22 (1.11–1.68)	0.1187	0.8881		
Child Behaviors							
Physical Activity Level ^2^	21.85 ± 12.17 (20.11–23.58)	21.67 ± 11.55 (19.45–23.89)	20.40 ± 12.69 1 (8.75–22.04)	0.8361	0.434		
Sleep (Hours/Night)	8.80 ± 1.39 (8.61–9.00)	8.57 ± 1.40 (8.30–8.84)	8.80 ± 1.25 (8.63–8.96)	1.2438	0.2891		
Fruit/Vegetable Servings/Day	4.39 ± 1.95 (4.11–4.67)	4.57 ± 2.22 (4.14–5.00)	4.47 ± 2.33 (4.16–4.77)	0.2308	0.7940		
Sugar-Sweetened Drink Servings/Day	1.03 ± 1.42 (0.83–1.23)	1.28 ± 1.61 (0.97–1.59)	1.20 ± 1.93 (0.95–1.45)	0.8642	0.4220		
Household Behaviors							
Family Meals/Week	13.98 ± 6.09 (13.11–14.85)	13.93 ± 5.39 (12.90–14.97)	12.40 ± 5.75 (11.65–13.15)	4.7139	0.0094	B	0.018
Family Meals/Week in Unhealthy Locations	2.19 ± 3.13 (1.74–2.63)	2.78 ± 3.36 (2.14–3.43)	2.40 ± 3.38 (1.97–2.84)	1.115	0.3287		
Family Meals/Week and Simultaneous Electronic Media Use	3.04 ± 2.93 (2.62–3.45)	3.04 ± 2.81 (2.50–3.58)	3.01 ± 2.78 (2.65–3.37)	0.0046	0.9954		
Home Environment							
Indoor Space and Supports for Physical Activity ^3^	3.95 ± 0.83 (3.83–4.06)	3.68 ± 0.94 (3.50–3.86)	3.78 ± 0.86 (3.67–3.89)	3.7343	0.0245		
Outdoor/Yard Space and Supports for Physical Activity ^3,4^	4.49 ± 0.64 (4.39–4.59)	4.41 ± 0.60 (4.29–4.54)	4.36 ± 0.78 (4.25–4.47)	1.566	0.2100		
Sleep Supports ^3^	4.48 ± 0.91 (4.35–4.61)	4.32 ± 0.99 (4.13–4.51)	4.40 ± 0.88 (4.28–4.51)	1.1199	0.3271		
Home Availability of Fruits/Vegetables (Servings/Household Member/Day)	6.64 ± 3.04 (6.21–7.08)	6.22 ± 2.79 (5.68–6.76)	6.43 ± 2.90 (6.05–6.80)	0.7357	0.4797		
Home Availability of Sugar-Sweetened Beverages (Servings/Household Member/Day)	3.99 ± 5.54 (3.19–4.78)	3.14 ± 4.29 (2.31–3.96)	3.08 ± 4.82 (2.46–3.71)	1.9168	0.1481		
Space and Supports for Family Meals ^3^	4.29 ± 0.80 (4.17–4.40)	4.24 ± 0.69 (4.10–4.37)	4.28 ± 0.72 (4.18–4.37)	0.1699	0.8438		
Weight Status							
Maternal BMI	27.52 ± 6.71 (26.56–28.48)	25.53 ± 6.55 (24.27–26.79)	26.00 ± 6.20 (25.19–26.80)	4.2595	0.0146		
Perception of Child Weight Status ^5^	3.78 ± 0.96 (3.64–3.92)	3.73 ± 1.06 (3.52–3.93)	3.73 ± 0.97 (3.61–3.86)	0.1639	0.8489		

^1^ Possible score = 0 to 49. ^2^ Possible score = 0 to 42. ^3^ Possible score = 1 to 5, higher scores indicate greater space/supports. ^4^ N = 161, N = 87, and N = 202 for No Work for Pay, Part-Time Employment, And Full-Time Employment, respectively. ^5^ Possible score = 1 to 7; age-appropriate, sex-matched silhouettes ranging from very underweight to very obese. ^6^ Significant pairwise comparisons: A= no work for pay vs. part-time employment; B= no work for pay vs. full-time employment; C= part-time employment vs. full-time employment.

**Table 3:** Maternal work impact clusters.

Work Impact Clustering Variables ^1^	Cluster 1: Enthusiastic Earners	Cluster 2: Indifferent Earners	Cluster 3: Strained Earners	ANOVA		Tukey’s Post Hoc Pairwise Comparisons for *p* ≤ 0.01 ^2^	Partial Eta-Squared
	**n = 130**	**n = 109**	**n = 97**	**F**	***p***		
	**Mean ± SD**	**Mean ± SD**	**Mean ± SD**	**df = 2, 333**			
	**(95% CI)**	**(95% CI)**	**(95% CI)**				
Work Motivation	4.32 ± 0.60 (4.21–4.42)	3.24 ± 0.86 (3.08–3.41)	3.98 ± 0.70 (3.84–4.13)	67.4222	<0.0001	ABC	0.288
Work Involvement	3.51 ± 0.80 (3.37–3.65)	3.03 ± 0.51 (2.94–3.13)	3.86 ± 0.71 (3.72–4.00)	37.6366	<0.0001	ABC	0.184
Work–Family Strains	1.76 ± 0.56 (1.66–1.86)	2.68 ± 0.76 (2.53–2.82)	3.59 ± 0.57 (3.48–3.71)	232.9855	<0.0001	ABC	0.583
Work–Family Gains	4.34 ± 0.47 (4.26–4.42)	3.28 ± 0.56 (3.18–3.39)	4.01 ± 0.50 (3.91–4.11)	132.6469	<0.0001	ABC	0.443
Work–Parenting Strains	1.94 ± 0.64 (1.83–2.05)	2.78 ± 0.82 (2.63–2.94)	3.69 ± 0.54 (3.58–3.79)	184.3504	<0.0001	ABC	0.525
Work–Parenting Gains	4.25 ± 0.53 (4.16–4.34)	3.20 ± 0.64 (3.08–3.32)	4.02 ± 0.56 (3.91–4.13)	106.2925	<0.0001	ABC	0.390

^1^ Five-point Likert Scales, ranging from 1 = strongly disagree to 5 = strongly agree; Cronbach alpha in order of clustering variables listed in the table: 0.78 (3 items), n/a (1 item), 0.91 (7 items), 0.91 (7 items), 0.90 (6 items), 0.84 (4 items). ^2^ Significant pairwise comparisons: A = Clusters 1 and 2; B = Clusters 1 and 3; C = Clusters 2 and 3.

**Table 4:** Sociodemographic characteristics compared by maternal work impact cluster.

Characteristic	Cluster 1: Enthusiastic Earners	Cluster 2: Indifferent Earners	Cluster 3: Strained Earners	ANOVA		Tukey Post Hoc Pairwise Comparisons for *p* ≤ 0.01 ^4^	Partial Eta-Squared
	**n = 130**	**n = 109**	**n = 97**	**F**	***p***		
	**Mean ± SD**	**Mean ± SD**	**Mean ± SD**	**df = 2, 333**			
	**(95% CI)**	**(95% CI)**	**(95% CI)**				
Age (Years)	39.40 ± 5.28 (38.48–40.32)	37.31 ± 6.53 (36.07–38.55)	36.59 ± 5.65 (35.45–37.73)	7.3418	0.0008	AB	0.042
Race/Ethnicity ^1^	0.57 ± 0.50 (0.48–0.66)	0.61 ± 0.49 (0.52–0.71)	0.75 ± 0.43 (0.67–0.84)	4.28	0.0146		
Education Level ^2^	2.53 ± 0.66 (2.42–2.65)	2.41 ± 0.70 (2.28–2.55)	2.58 ± 0.59 (2.46–2.70)	1.7753	0.171		
# Children <18 Years in Household	2.10 ± 1.01 (1.92–2.28)	1.93 ± 0.88 (1.76–2.09)	2.14 ± 0.97 (1.95–2.34)	1.5463	0.2146		
# Parents in Household	1.78 ± 0.41 (1.71–1.86)	1.75 ± 0.43 (1.67–1.83)	1.79 ± 0.41 (1.71–1.88)	0.2905	0.7481		
Maternal Household, Parenting, and Community Activities (Hours/Day)	3.28 ± 1.71 (2.98–3.58)	3.21 ± 1.94 (2.84–3.58)	3.02 ± 1.72 (2.67–3.36)	0.62	0.5379		
Spouse/Partner	n = 102	n = 82	n = 77				
Spouse/Partner Employment ^3^	1.75 ± 0.64 (1.62–1.87)	1.68 ± 0.68 (1.53–1.83)	1.73 ± 0.60 (1.59–1.86)	0.2194	0.8031		
Spouse/Partner Education Level ^2^	2.29 ± 0.80 (2.14–2.45)	2.04 ± 0.91 (1.84–2.24)	1.96 ± 0.91 (1.75–2.17)	3.7068	0.0259		

^1^ Coded as 0 = white; 1 = non-White. ^2^ Coded as 1 = high school or less; 2 = some post-secondary education (e.g., college, technical school); 3 = baccalaureate degree or higher. ^3^ Coded as 0 = does not work for pay; 1 = works part-time for pay; 2 = works full-time for pay. ^4^ Significant pairwise comparisons: A = Clusters 1 and 2; B = Clusters 1 and 3; C = Clusters 2 and 3.

**Table 5:** Employment characteristics compared by maternal work impact cluster.

Characteristic	Cluster 1: Enthusiastic Earners	Cluster 2: Indifferent Earners	Cluster 3: Strained Earners	ANOVA		Tukey’s Post Hoc Pairwise Comparison for *p* ≤ 0.01 ^2^	Partial Eta-Squared
	**n = 130**	**n = 109**	**n = 97**	**F**	***p***		
	**Mean ± SD**	**Mean ± SD**	**Mean ± SD**	**df = 2, 333**			
	**95% CI**	**95% CI**	**95% CI**				
Number of Jobs	1.15 ± 0.42 (1.08–1.23)	1.13 ± 0.41 (1.05–1.21)	1.14 ± 0.38 (1.07–1.22)	0.12	0.89		
*% With 1 Job*	*86.92*	*89.91*	*86.6*				
Days/week Worked	4.91 ± 0.78 (4.77–5.04)	4.75 ± 1.10 (4.54–4.96)	4.80 ± 1.04 (4.60–5.01)	0.80	0.4492		
Work Outside Home Days/Week	3.48 ± 2.26 (3.09–3.87)	3.12 ± 2.27 (2.69–3.55)	3.62 ± 1.99 ( 3.22–4.02)	1.4645	0.2327		
Pay is Set Salary ^1^	0.45 ± 0.50 (0.36–0.53)	0.36 ± 0.48 (0.27–0.45)	0.45 ± 0.50 (0.35–0.55)	1.2715	0.2818		
*% With Set Salary*	*44.62*	*35.78*	*45.36*				
Hours/Week Worked	33.24 ± 11.70 (31.21–35.27)	28.97 ± 13.22 (26.46–31.48)	27.33 ± 14.56 (24.40–30.26)	6.33	0.002	AB	0.037
Extra Hours/Week Worked	2.12 ± 4.25 (1.38–2.85)	1.68 ± 3.34 (1.05–2.31)	3.66 ± 4.58 ( 2.74–4.58)	6.63	0.0015	BC	0.038
Hours/Week Worked ± Extra Hours/Week Worked	35.35 ± 13.29 (33.05–37.66)	30.65 ± 13.99 (28.00–33.31)	30.99 ± 15.48 (27.87–34.11)	4.11	0.0173		
Commute Minutes/Week to/from Work	109.54 ± 140.75 (85.11–133.96)	105.92 ± 120.02 (83.13–128.70)	158.45 ± 154.90 (127.23–189.67)	4.5994	0.0107		
Works Weekends ^1^	0.14 ± 0.35 (0.08–0.20)	0.28 ± 0.45 (0.19–0.36)	0.25 ± 0.43 (0.16–0.33)	3.79	0.0236		
*% Works Weekends*	*13.85*	*27.52*	*24.74*				
Works Same Days/Week ^1^	0.93 ± 0.25 (0.89–0.97)	0.89 ± 0.31 (0.83–0.95)	0.91 ± 0.29 (0.85–0.97)	0.62	0.5409		
*% Works Same Days/Week*	*93.08*	*88.99*	*90.72*				
Works Same Hours/Day ^1^	0.95 ± 0.23 (0.91–0.99)	0.92 ± 0.28 (0.86–0.97)	0.91 ± 0.29 (0.85–0.97)	0.69	0.5042		
*%Works Same Hours/Day*	*94.62*	*91.74*	*90.72*				
Starts Work at Standard Morning Hours ^1^	0.93 ± 0.25 (0.89–0.97)	0.88 ± 0.33 (0.82–0.94)	0.90 ± 0.31 (0.84–0.96)	0.91	0.4048		
*% Starts Work at Standard Morning Hours*	*93.08*	*88.07*	*89.69*				
Ends Work at Standard Evening Hours ^1^	0.92 ± 0.28 (0.87–0.96)	0.93 ± 0.26 (0.88–0.98)	0.92 ± 0.28 (0.86–0.97)	0.05	0.9473		
*% Ends Work at Standard Evening Hours*	*91.54*	*92.66*	*91.75*				

^1^ Coded as 0 = no; 1 = yes. ^2^ Significant pairwise comparisons: A = Clusters 1 and 2; B = Clusters 1 and 3; C = Clusters 2 and 3.

**Table 6:** Behavior, home environment, and weight status assessments by maternal work impact cluster.

Assessment	Cluster 1: Enthusiastic Earners	Cluster 2: Indifferent Earners	Cluster 3: Strained Earners	ANOVA		Tukey’s Post Hoc Pairwise Comparisons for *p* ≤ 0.01 ^6^	Partial Eta-Squared
	**n = 130**	**n = 109**	**n = 97**	**F**	***p***		
	**Mean ± SD**	**Mean ± SD**	**Mean ± SD**	**df = 2, 333**			
	**(95% CI)**	**(95% CI)**	**(95% CI)**				
Maternal Behaviors							
Physical Activity Level ^1^	18.68 ± 13.01 (16.42–20.93)	14.89 ± 13.48 (12.33–17.45)	20.14 ± 11.83 (17.76–22.53)	4.7134	0.0096	C	0.028
Sleep (Hours/Night)	7.25 ± 0.97 (7.08–7.42)	7.11 ± 1.16 (6.89–7.33)	7.25 ± 1.23 (7.00–7.49)	0.5556	0.5743		
Fruit/Vegetable Servings/Day	4.44 ± 2.06 (4.08–4.80)	3.92 ± 2.04 (3.53–4.31)	4.96 ± 2.13 (4.53–5.39)	6.4015	0.0019	C	0.037
Sugar-Sweetened Drink Servings/Day	1.10 ± 1.77 (0.79–1.41)	1.22 ± 2.12 (0.82–1.63)	2.09 ± 2.15 (1.66–2.53)	7.6099	0.0006	BC	0.044
Child Behaviors							
Physical Activity Level ^2^	21.58 ± 13.22 (19.29–23.88)	20.72 ± 11.97 (18.44–22.99)	19.84 ± 11.56 (17.51–22.16)	0.5604	0.5715		
Sleep (Hours/Night)	8.94 ± 1.02 (8.77–9.12)	8.86 ± 1.35 (8.60–9.11)	8.28 ± 1.49 (7.98–8.58)	8.2873	0.0003	BC	0.047
Fruit/Vegetable Servings/Day	4.61 ± 2.33 (4.21–5.02)	4.00 ± 2.14 (3.59–4.41)	4.91 ± 2.34 (4.44–5.38)	4.3665	0.0134		
Sugar-Sweetened Drink Servings/Day	0.95 ± 2.00 (0.60–1.30)	0.83 ± 1.04 (0.63–1.02)	2.03 ± 2.05 (1.62–2.45)	14.5457	<0.0001	BC	0.08
Household Behaviors							
Family Meals/Week	13.76 ± 5.68 (12.78–14.75)	12.35 ± 5.69 (11.27–13.43)	12.31 ± 5.57 (11.19–13.43)	2.5575	0.079		
Family Meals/Week in Unhealthy Locations	1.70 ± 2.82 (1.21–2.19)	2.44 ± 3.27 (1.82–3.06)	3.72 ± 3.82 (2.95–4.49)	10.5948	<0.0001	BC	0.06
Family Meals/Week and Simultaneous Electronic Media Use	2.39 ± 2.78 (1.91–2.87)	3.23 ± 2.92 (2.68–3.78)	3.63 ± 2.48 (3.13–4.13)	6.1026	0.0025	B	0.035
Home Environment							
Indoor Space and Supports for Physical Activity ^3^	4.01 ± 0.89 (3.85–4.16)	3.44 ± 0.88 (3.28–3.61)	3.74 ± 0.78 (3.59–3.90)	12.9196	<0.0001	AC	0.072
Outdoor/Yard Space and Supports for Physical Activity ^3,4^	4.54 ± 0.52 (4.45–4.64)	4.15 ± 0.82 (3.97–4.32)	4.10 ± 0.74 (3.94–4.26)	13.0154	<0.0001	AB	0.072
Sleep Supports ^3^	4.68 ± 0.72 (4.55–4.80)	4.17 ± 1.08 (3.96–4.37)	4.20 ± 0.85 (4.02–4.37)	12.5224	<0.0001	AB	0.07
Home Availability of Fruits/Vegetables (Servings/Household Member/Day)	7.16 ± 2.92 (6.65–7.66)	5.95 ± 3.07 (5.37–6.53)	5.76 ± 2.27 (5.30–6.22)	8.6277	0.0002	AB	0.049
Home Availability of Sugar-Sweetened Beverages (Servings/Household Member/Day)	2.95 ± 4.83 (2.12–3.79)	3.04 ± 4.95 (2.10–3.98)	3.36 ± 4.07 (2.54–4.18)	0.2202	0.8025		
Space and Supports for Family Meals ^2^	4.58 ± 0.54 (4.49–4.68)	4.06 ± 0.80 (3.91–4.21)	4.06 ± 0.66 (3.93–4.19)	24.6082	<0.0001	AB	0.129
Weight Status							
Maternal BMI	25.56 ± 5.61 (24.59–26.53)	26.70 ± 6.95 (25.38–28.02)	25.29 ± 6.40 (24.00–26.58)	1.5002	0.2246		
Perception of Child Weight Status ^5^	3.65 ± 0.90 (3.50–3.81)	3.76 ± 1.10 (3.55–3.97)	3.79 ± 1.00 (3.59–4.00)	0.6319	0.5322		

^1^ Possible score = 0 to 49. ^2^ Possible score = 0 to 42. ^3^ Possible score = 1 to 5, higher scores indicate greater space/supports. ^4^ N = 117, N = 87, and N = 85 for Clusters 1, 2, and 3, respectively. ^5^ Possible score = 1 to 7; age-appropriate, sex-matched silhouettes ranging from very underweight to very obese. ^6^ Significant pairwise comparisons: A = Clusters 1 and 2; B = Clusters 1 and 3; C = Clusters 2 and 3.

## Data Availability

No new data were created or analyzed in this study. Data sharing is not applicable to this article.
